# Duration of unemployment and depression: a cross-sectional survey in Lithuania

**DOI:** 10.1186/1471-2458-6-174

**Published:** 2006-07-05

**Authors:** Mindaugas Stankunas, Ramune Kalediene, Skirmante Starkuviene, Violeta Kapustinskiene

**Affiliations:** 1 Department of Social Medicine, Kaunas University of Medicine, A. Mickeviciaus St. 9, LT-44307 Kaunas, Lithuania; 2 Faculty of Public Health, Kaunas University of Medicine, A. Mickeviciaus St. 9, LT-44307 Kaunas, Lithuania

## Abstract

**Background:**

In spite of a growing economy, unemployment is still a severe socio-economic problem in Lithuania. Nonetheless, no studies have been performed about the associations between unemployment and mental health in Lithuania. The aim of this study was to evaluate the associations between unemployment duration and depression in Lithuania.

**Methods:**

The data was collected in a cross-sectional study in 2005. There were 429 filled-in questionnaires received (53.6% response rate) from unemployed persons registered with the Kaunas Labour Market Office. The severity of depression symptoms was evaluated using the Beck Depression Inventory (BDI). Logistic regression was used to estimate the risk factors for occurrence of depression. Sex, age, place of residence, marital status, education, income and practiced religion were the independent variables. Long-term unemployment was defined as lasting a duration of 12 months or more.

**Results:**

The findings showed that long-term unemployed persons had more episodes of a depressive mood in the past 12 months in comparison with the group of the short-term unemployed. In addition, the BDI score mean was higher among the long-term unemployed compared with the short-term unemployed (10.1 ± 8.8 and 14.2 ± 9.5 respectively, p < 0.001). It was estimated that the duration of unemployment and BDI score had a positive correlation (r = 0.1968, p < 0.001). Among the short-term unemployed, the risk of depression increased significantly when the person was female, had an older age and had experienced more episodes of unemployment. Among the long-term unemployed, an older age was the risk factor for development of depression. However, higher education and income were the factors that significantly decreased the risk of developing depression for-short term as well as for long-term unemployed.

**Conclusion:**

The results indicated that depression is a severe problem in the unemployed population. Depression is more elevated among the long-term unemployed. This leads to arguing for common efforts in providing needed social support and health care to reduce the effects of unemployment on mental health.

## Background

Employment, in most societies, is one of the factors used to describe the economic and social situation of the country. Employment facilitates the fulfilment of socially defined needs in two ways – social identity in a society where work is the norm and the means for economic participation in a society where work is the main source of private economic resources [1]. Dahlgren et al. mention that the loss of employment is one of the factors that has considerable influence on health status [2]. Many previous studies have reported a relationship between unemployment and ill health [3-6]. A recent study from Finland indicated that current unemployment was associated with the major depressive episodes in general population [7]. *HEALTH 21*, a document of the World Health Organization, stresses the importance of tackling social inequalities in health [8]. This issue is especially important in countries that are undergoing rapid social, economic and political reforms. Such changes influence the social, demographic and health situation of a population.

Fifteen years have passed since Lithuania declared independence in 1990. The newly independent country faced many challenges in the transformation of political, social and economic systems. The transition from a planned to a market economy caused an increase in competition, rise of private capital and changes in the labour market that involved growing unemployment. Until the early 1990s, the prevalence of joblessness in Lithuania was very low (about 1% of the working age population). The unemployment rate gradually increased during the 1993–2001 period. In 2001, the unemployment rate reached its highest, ever-reported level of 16.4% [9]. Since 2002, joblessness started to decrease; nevertheless, the unemployment rate was still 8.2% in Lithuania in 2005 [10]. In spite of that, there is an inadequate social support system for unemployed persons in Lithuania. Only 16% of registered unemployed persons receive unemployment benefits. More than 28% of these persons are long-term unemployed [11].

The prevalence of mental diseases increased by 34.5% during the period of 1998–2003, amounting to 2,653.6 cases per 100,000 persons in the population. Various epidemiological studies show that about one of every seven people in Lithuania needs psychological, psychotherapeutic or psychiatric counselling [12]. Moreover, suicide rates in Lithuania are among the highest in the world. For example, the suicide rate, standardized by age (using the European standard population), was 38.9 suicides per 100,000 people in the population (70.0 among males and 12.7 among females per 100,000) in 2004 [13]. Until recently, only a few efforts have been made to investigate the associations between mental health and unemployment in Lithuania [14,15]. Therefore, studies on the impact of unemployment on health should be of major importance for improving mental health among different social groups in Lithuania.

The aim of this study was to evaluate the associations between unemployment duration and depression in Lithuania.

## Methods

This study was performed in Kaunas, the second largest city located in the central part of Lithuania. The city is an industrial and commercial centre, where textiles, processed food, metal goods, and machinery are produced. The population of the city (as per a 2004 estimate) is 366,652. The official rate of unemployment among the working age population of Kaunas in May 2005 was 3.5%. The analysis herein covers the officially registered unemployed persons in Kaunas. The International Labour Organization defines a long-term unemployed person as one who is unemployed for 12 months or more [9].

This is a cross-sectional study. Questionnaires were distributed to randomly selected unemployed persons who attended the Kaunas Labour Market Office (Labour Office, hereafter) in February-April of 2005. The respondents were informed about the aims and process for the study. The administration of the Labour Office and the Bioethics Centre at Kaunas University of Medicine granted the permission to perform this study.

The total number of unemployed in Kaunas at the time of this study was 10,027 persons. The questionnaires were distributed to 800 unemployed persons attending Labour Office. The chosen number of respondents was twice the size of the calculated representative sample (370 unemployed were needed to achieve a 95% confidence level), because we expected a 50% response rate. The random sample of unemployed from Kaunas was drawn from the Labour Office database. The random sample was generated of 800 records of unemployed: the selection criterion was the randomly chosen days of registration at Labour Office. All unemployed persons, who were registered during the selected days, were invited to participate in this survey. According to rules of the Labour Office, each unemployed person has to attend special meetings at the given time. The questionnaires were distributed to selected respondents during these meetings. The total number of returned questionnaires was 429 (a response rate – 53.6%). The investigated study sample was representative to general population of unemployed in terms of main demographic characteristics (gender and age).

Two methods were used for the measurement of depression: 1) self-reported depression and 2) Beck Depression Inventory.

Self-reported depression was measured according to the responses to the following multiple-choice questions: *Did you have a bad (depressive) mood in the past 12 months? How has the occurrence of bad (depressive) moods changed after you became unemployed? *And, *Have you used sedative drugs in past 30 days?*

The Beck Depression Inventory – BDI hereafter – [16] is a 21-item questionnaire, widely used to measure the severity of depressive symptoms. It contains 21 items, asking about cognitive, motivational, affective and behavioural symptoms of depression [17]. Each item is scored on a 0–3 point scale, indicating the severity/persistence of a symptom, where 0 indicates that a symptom is absent and 3 indicates that a symptom is severe or persistent. BDI scores range from 0 to 63. Severity scores are interpreted as follows: 0–9 minimal, 10–16 mild, 17–29 moderate and 30–63 severe [18]. Depression was considered if the BDI score was 15 and more [19].

Data were computed, coded and analysed using the Statistical Package for the Social Sciences for Windows, Version 11.0 (SPSS Inc.) and Microsoft Excel 2000. The following statistical analyses were applied: 1) descriptive statistics; 2) logistic regression.

The statistical significance of difference between two groups was assed using a two-tailed Student (t) test for continuous variables. Correlation (r) between duration of unemployment (in months) and BDI score was assessed.

For evaluation of the impact of explanatory variables on analysed event, (binary dependent variable) *Enter *model of logistic regression was used. Dependent variable was depression (was depressed or not, according to BDI test). Sex, age, education, residence location, marital status, income, practiced religion, duration, and episodes of unemployment were used as independent variables. Risk was measured using the odds ratio (OR), calculating the 95% confidence interval (CI). Differences in results at the p < 0.05 level were considered statistically significant.

## Results

Of the 429 respondents, 153 (35.7%) were males and 276 (64.3%) females. The distribution of respondents by age was the following: under 25 years (14.5%), 25–34 years (24.5%), 35–44 years (28.0%), 45–54 years (25.9%) and 55–64 years (7.2%). The prevalence of main socio-demographic characteristics by type of unemployment is presented in Table 1. It demonstrates that the short-term unemployed were younger, more educated and resided in urban areas in comparison with the long-term unemployed respondents.

The prevalence of self-reported depression was evaluated by the duration of unemployment (Table 2). The findings show that persons with long-term unemployment had more episodes of a depressive mood in the past 12 months than did those in the short-term unemployed group. Changes in the occurrence of depressive moods after the onset of unemployment were measured as well. According to the responses, significantly more respondents in the short-term group declared that they had not felt considerable changes (p < 0.05). More than the half of the long-term unemployed indicated that depression became more common after unemployment. This group expressed more use of sedative drugs as well but the difference was not statistically significant.

The BDI was used to measure the severity of depressive symptoms. The means of the BDI Index and the distribution of the levels of depression symptoms by duration of unemployment are presented in Table 3. Based on these findings, it is claimed here that the long-term unemployed express more depression symptoms than do the short-term unemployed. In addition, the BDI score mean was higher among women than it was among men (12.7 ± 9.7 and 10.5 ± 8.5 respectively, p < 0.05).

The correlation between the length of unemployment and the BDI Index was calculated. The observed association was significant but not considerably so: the correlation coefficient was 0.1968 (p < 0.001).

Logistic regression was used to estimate the risk factors involved in the occurrence of depression (Table 4). Among the short-term unemployed, the risk of depression significantly increased for females (OR = 2.78), for those who were older (OR = 1.56) and had more episodes of unemployment (OR = 1.49). However, higher education (OR = 0.76) and income (OR = 0.68) were the factors that significantly decreased the risk of depression. In the case of the long-term unemployment, older age (OR = 1.43) was the significant risk factor for developing depression. Higher education (OR = 0.61) and higher income (OR = 0.71) however, decreased the risk of developing depression in the long-term unemployed group. For all respondents, being female (OR = 1.62), older age (OR = 1.48) and bigger number of unemployment times (OR = 1.28) increased the risk of depression. While opposite effect was observed with the education (OR = 0.75) and higher income (OR = 0.69).

## Discussion

Prior to proceeding with the discussion on the results of this study, certain important methodological issues require explanation.

First is the rate of unemployment registration. The Lithuanian Labour Market (LLM, hereafter) collects and analyses the movement of the labour force in Lithuania. Study sample fully represented the general unemployed population in Kaunas in terms of gender and age. According to official data of LLM, 60.5% of unemployed were females and 39.5% were males (in the study sample – 64.3% and 35.7%, respectively). Distribution according the age was also similar, except 55–64 years age group (11.9% in the total unemployed population vs. 7.2% in the study sample, p < 0.05) [11]. However, the official unemployment data is likely to be an underestimation of the actual number of jobless individuals, who are either not registered with the LLM or working illegally. This state register does not include persons seeking jobs through private labour agencies. Various cross-sectional studies performed in Lithuania suggest that rate of unemployment is 2–2.5 times higher than that reported by official statistics [20,21].

Secondly, relevant to the first issue, the results are possibly limited in part by the type of respondents selected for this study. Only those unemployed persons, who were registered with the Labour Office, participated as respondents. The opinion here is that these respondents are probably more socialized and less "socially marginal" than the jobless individuals who do not register with their local labour office. Other studies on unemployment have referenced such social marginalization as indicating a greater probability that such persons engage in asocial behaviour [22]. Thus, it can be predicted that these people also have mental health problems. This study does not assess non-respondents.

Finally, this study is not longitudinal. The scope of this study did not permit follow-up of the respondents during their full unemployment period for an evaluation regarding changes in their mental health.

Unemployment, as a socioeconomic factor, is relatively new in Lithuanian society. The first cases of unemployment were only registered after the collapse of the Soviet Union in 1991. Previously, according to USSR legislation, not only did the government guarantee work but work was also an obligation to society. Therefore, each graduate was delegated to some pre-selected job position. Since the beginning of the political and economic reforms in 1989 and after the declaration of independence, circumstances in Lithuania changed dramatically. The population of the country was exposed to fundamental and unfamiliar economic, political and social changes [23]. The transition from a planned to market economy had a considerable impact on the employment situation in Lithuania. By late 1992, the unemployment rate started to increase and by the end of 1996, it had climbed to 7%. In the summer of 1997, the Government of Lithuania passed a law that increased the minimum wage from 330 to 400 LTL (the Lithuanian currency, the *litas *was then equal to 0.25 USD). By virtue of this regulation, the workforce became more expensive and therefore, unemployment could suddenly increase. Indeed, the peak in the unemployment rate reached 16.4% in early 2001 [9]. In recent years, unemployment started to decrease; however, Lithuania is still facing a rate that is higher than the average in other European Union countries [10]. As previously mentioned, more than 28% of those registered as unemployed have been jobless over the long-term [11]. For this study, the effort was to evaluate the occurrence of depression according to the duration of unemployment and special interest was paid to the group of the long-term unemployed.

Previous sociological studies on unemployment in Lithuania indicated that long-term unemployment caused people to lose faith in possible re-employment. Therefore, these people were more likely to seek unofficial or short-term/seasonal jobs instead of long-term, official positions [24]. The possible cause of such behaviour among the unemployed includes passiveness, lack of motivation and an unwillingness to change. The results of this study emphasize that self-reported depressive moods occurred more often among persons, unemployed for a long-term rather than a short-term. These findings support the link between unemployment duration and poor psychological health: the longer a person is unemployed, the worse health becomes. Winefield concluded that the damage to mental well-being and the intensity of psychological distress depend greatly on the duration of unemployment [25]. The results of a Japanese study showed that depressive symptoms were stable during the first year of unemployment but observed a sharp increase after one year. The authors of this study suggest that this might have been due to the financial support provided by unemployment benefits. When the right to unemployment benefits expired, mood disorders occurred [26]. This study did not assess the link between depression and unemployment benefits. The reasons for this were as follows: 1) only a small proportion of the unemployed (16% of those registered by the LLM) receive unemployment benefits and 2) this benefit is too small (approximately 90 USD per month) to adequately cover minimal living expenses. These aspects determined the choice to measure the importance of general household income per capita per month. The logistic regression analysis showed that a higher income reduces the risk of depression. The results from a cross-sectional study of seven post-soviet countries (including Lithuania) substantiate that material well-being has considerable impact on self-reported health [27]. The entirely likely prediction is that unemployment benefits did not play a crucial role in the occurrence of depression symptoms among job seekers. The economic situation of the entire household plays a much more important role.

To obtain results on depression that are more confident, the Beck Depression Inventory was used. It is necessary to note that this methodology was used in a number of previous studies on this topic [28-30]. The listed studies show that the means of BDI were higher among the unemployed in comparison with the employed. Moreover, the BDI scores in the referenced studies were lower than were those, found among out-of-work Lithuanians [28,29]. One possible explanation is that the general population of Lithuania tends to suffer more from depression. The extremely high suicide rates express this indirectly [31]. Many authors conclude that the dramatic changes in the cultural, economic and social life of the society are risk factors for suicide [32]. Growing unemployment could be listed as one of the most considerable changes in the newly independent state. Several studies have revealed increased rates of suicide during periods of economic recession and high unemployment [33,34]. Nevertheless, during the period of the most intensive increase in unemployment, suicide rates were decreasing in Lithuania. A similar phenomenon was reported in Latvia, suggesting that the relationship between unemployment and suicide is very complex and atypical both in Latvia and in Lithuania. Other factors appear to play roles that are more important in influencing the levels of suicide in these countries [35]. It might be that the association between unemployment and suicide exists due the strong persistence of a *culture of poverty *in Lithuania, especially among jobless individuals. American anthropologist Oscar Lewis introduced the culture of poverty theory in 1966. Here, it is suggested that this theory is relevant in discussing the links between unemployment and depression. According to this theory, poor people have a strong feeling of marginality, helplessness, dependency and not belonging. These feelings generate a way of life that constitutes a *culture of poverty *[36]. Some connections between a *culture of poverty *and unemployment could be identified. Unemployment has a particular impact on the personality: additional worries, irritability and decreased attention and ability to concentrate. These lead to an uncertain life conduct, a crisis about the values that had previously guided behaviour and the development and embedding of neurotic reactions [37-39]. Additionally, unemployed persons start to feel shame about belonging to this social category [40]. Subsequently, unemployment leads to negative lifestyle changes [41]. Unemployed persons are more likely to engage in drinking alcohol [42], smoking [43], drug use, suicide intentions and crime [44]. The studies coming from Lithuania argue that there are some elements of the *culture of poverty *among the unemployed in the country. Jatuliene et al. evaluated attitudes of the unemployed in Lithuania regarding their quality of life. According to this referenced study, the majority of persons who had lost their jobs evaluated their health and quality of life as poor or very poor. Moreover, more then 70% of respondents had very pessimistic feelings regarding their futures [15]. Furthermore, during the most rapid increase of unemployment, changes in the age structure of the unemployed were observed. During the period of 1998 – 2001, there was a sharp increase in the unemployment rate among people, aged 50–60 years [45]. The problem of unemployment among this age group of the population increased because a majority of people of pre-retirement or retirement age were forced to leave their jobs [46]. Possibly, the frustration of losing work or an absence of work was the factor influencing higher suicides rates for this age group [13]. Also of note is that the value system of the Soviet Union still had a strong influence on the 50–60 year old population. This value system in the former society of Lithuania held that having no job was very shameful, stigmatic and unacceptable. Unemployed individuals of this generation might have very strong feelings of powerlessness, inferiority and personal unworthiness. According to Lewis, these are the key elements of the *culture of poverty*. Here, it is predicted that these factors could have influenced the expressed depression and high suicide rates among the unemployed [36]. It necessary to mention, that intensive scientific debates go on validity of the *culture of poverty *[47]. There are many studies which do not support link "unemployment – culture of poverty – poor mental health" [48,49]. For instance, Platt's study showed, that people living in places with low levels of unemployment have been founded to be more likely to commit suicide than people living elsewhere [49,50]. As well, an ecological study in England, examining trends in the 1980s, showed that places with the greatest increases in unemployment had the smallest increases in suicide [51].

The findings of this study and the results of the referenced studies lead to speculate about the existence of a specific phenomenon – a *culture of poverty *– among the unemployed in Lithuania. For an assessment of the existence of a link between a *culture of poverty *and unemployment and its effect on mental health, further studies should be performed in Lithuania. The attention needs to be on an assessment of the effect of unemployment on an individual level by performing qualitative investigations as well. Future studies should provide a better understanding of mental health and cover aspects such as sense of coherence, shame and ability to cope. Meanwhile, this study can serve as the background research for an in-depth analysis of unemployment and mental health associations in Lithuania.

## Conclusion

This study adds to the growing evidence of associations between unemployment and ill mental health. Results indicate that depression is more prevalent among the long-term unemployed. Being female, older age, and growing number of unemployment episodes has significant effect on development of depression, while having higher education and higher income reduce the risk. The fact that considerable proportion of unemployed people experience long-term unemployment makes this problem even more severe. Collective actions should be facilitated to address the challenges of psychological and social adaptability in cases of unemployment. Improvement could be achieved with strong pressure placed on the Government, municipal authorities and health services to take action regarding the health of the unemployed. Among the many opportunities to achieve this goal is an investment in human capital, redistributive policies and the ensuring of comprehensive access to health care. Mental health of the unemployed should receive serious attention in health policy development in Lithuania. Despite limited resources, mental health could be improved, not only through health care services but also, through social reforms.

## Competing interests

The author(s) declare that they have no competing interests.

## Authors' contributions

MS designed the study, performed the data analysis, drafted and revised the manuscript. RK participated in the initial study design and revision of the article. SS performed the data analysis and revised the article. VK collected the data and revised the manuscript. All authors read and approved the final version of this manuscript.

## Pre-publication history

The pre-publication history for this paper can be accessed here:



## Figures and Tables

**Table 1:** Socio-demographic characteristics of respondents

**Variable**	**Unemployment Duration (%/n)**		**t (p)**
		
	**Short-term N = 234**	**Long-term N = 195**	
**Gender**			
Males	34.2/80	37.4/73	0.69 (sn)
Females	65.8/154	62.6/122	0.69 (sn)
**Age group**			
16–24	19.2/45	8.7/17	3.21 (p < 0.01)
25–34	27.4/64	21.0/41	1.55 (sn)
35–44	25.2/59	31.3/61	1.40 (sn)
45–54	21.8/51	30.8/60	2.11 (p < 0.05)
55–64	6.4/15	8.2/16	0.71 (sn)
**Education level**			
Primary	2.1/5	1.0/2	0.93 (sn)
Incomplete secondary	4.3/10	9.2/18	1.99 (p < 0.05)
Secondary – professional	23.1/54	34.4/67	2.58 (p < 0.01)
Secondary	29.1/68	25.1/49	0.93 (sn)
College level	20.1/47	19.0/37	0.29 (sn)
University degree	21.4/50	11.3/22	2.88 (p < 0.01)
**Marital status**			
Single	26.1/61	23.6/46	0.60 (sn)
Cohabitation without marriage	4.3/10	9.7/19	2.16 (p < 0.05)
Married	49.6/116	46.7/91	0.60 (sn)
Divorced	17.5/41	17.9/35	0.11 (sn)
Widowed	2.6/6	2.1/4	0.34 (sn)
**Residence area**			
Urban	91.0/213	84.6/165	2.01 (p < 0.05)
Rural	9.0/21	15.4/30	2.01 (p < 0.05)

n – number of observed persons

p – significance level comparing short-term and long-term unemployment groups

sn – statistically non-significant

t – Student (t) criteria

**Table 2:** Self-reported depression

**Variable**	**Unemployment Duration (%/n)**		**t (p)**
		
	**Short-term N = 234**	**Long-term N = 195**	
**Depression (in past 12 months)**			
Never	15.8/37	16.4/32	0.17 (sn)
Sometimes	50.4/118	32.8/64	3.75 (p < 0.001)
Often	29.5/69	39.5/77	2.17 (p < 0.05)
Always	4.3/10	11.3/22	2.67 (p < 0.01)
**Depression (after unemployment)**			
Do not feel at all	14.1/33	11.8/23	0.71 (sn)
No significant changes	43.2/101	33.8/66	2.01 (p < 0.05)
Feel more often	39.7/93	51.8/101	2.52 (p < 0.05)
Feel less	3.0/7	2.6/5	0.25 (sn)
**Use of sedative drugs**			
At least once in the last 30 days	19.2/45	25.6/50	1.58 (sn)

n – number of observed persons

p – significance level comparing short-term and long-term unemployment groups

sn – statistically non-significant

t – Student (t) criteria

**Table 3:** Beck Depression Inventory Index means and levels of depression symptoms

**Variable**	**Unemployment Duration (%/n) (m ± SD)**		**t (p)**
		
	**Short-term N = 234**	**Long-term N = 195**	
BDI Index mean	10.1 ± 8.8	14.2 ± 9.5	4.60 (p < 0.001)
**Levels of depression symptoms**			
Minimal	57.7/135	35.9/70	4.62 (p < 0.001)
Mild	20.1/47	25.6/50	1.36 (sn)
Moderate	18.4/43	32.3/63	3.32 (p < 0.001)
Severe	3.8/9	6.2/12	1.08 (sn)
Depression with BDI 14/15 cut-off point	27.4/64	45.6/89	3.95 (p < 0.001)

m – mean

SD – standard deviation

n – number of observed persons

p – significance level comparing short-term and long-term unemployment groups

sn – statistically non-significant

t – Student (t) criteria

**Table 4:** Risk of depression among the unemployed, logistic regression analysis

**Variable**	**Short-term unemployed N = 234**		**Long-term unemployed N = 195**		**All respondents N = 429**	
	**OR**	**95% CI**	**OR**	**95% CI**	**OR**	**95% CI**

Being female	2.78	1.32–5.86	1.08	0.55–2.12	1.62	1.01–2.62
Older age (each age group)	1.56	1.16–2.08	1.43	1.04–1.97	1.48	1.20–1.82
Education (higher level of education)	0.76	0.57–1.01	0.61	0.56–0.99	0.75	0.62–0.92
Urban residence	1.71	0.52–5.62	0.61	0.26–1.46	0.83	0.42–1.70
Living without a partner	1.27	0.65–2.48	1.63	0.84–3.14	1.48	0.94–2.32
Higher income	0.68	0.54–0.86	0.71	0.56–0.88	0.69	0.59–0.81
Unemployment duration (each additional month)	1.02	0.91–1.14	1.00	0.99–1.00	1.00	0.99–1.01
Number of unemployment periods (each additional time)	1.49	1.14–1.95	1.14	0.91–1.43	1.28	1.07–1.52
Regularly practiced religion	1.89	0.97–3.67	0.84	0.44–1.60	1.21	0.78–1.90

OR – odds ratio

CI – confidence interval
